# Comparative evaluation of the Thermo fisher TaqPath™ COVID-19 combo kit with the Cepheid Xpert® Xpress SARS-CoV-2 assay for detecting SARS-CoV-2 in nasopharyngeal specimens

**DOI:** 10.1186/s12879-021-06347-6

**Published:** 2021-06-30

**Authors:** Paul A. Granato, Simon R. Kimball, Brenda R. Alkins, Deirdre C. Cross, Melissa M. Unz

**Affiliations:** 1 Microbiology Department, Laboratory Alliance of Central New York, Liverpool, New York USA; 2 Department of Pathology, SUNY Upstate Medical University, Syracuse, New York USA; 3 Genetic Sciences Division, Thermo Fisher Scientific, Carlsbad, California USA

**Keywords:** TaqPath combo kit, Xpert Xpress SARS-CoV-2 assay, PCR, SARS-CoV-2, COVID-19, Gene amplification tests, Sanger sequencing

## Abstract

**Purpose:**

With over 50 SARS-CoV-2 gene amplification assays that have been EUA cleared with minimal experimental validation performed, it is likely that not all of these assays are comparable in their ability to detect SARS-CoV-2 in clinical specimens. Thermo Fisher Scientific is a relatively new company in the molecular diagnostics field and the purpose of this study was to compare the performance of the Thermo Fisher TaqPath™ Combo Kit with an established test, the Cepheid Xpert® Xpress SARS-CoV-2 assay, for its ability to detect SARS-CoV-2 in nasopharyngeal specimens.

**Methods:**

A total of 300 randomly selected nasopharyngeal specimens were evaluated and tested by the TaqPath and GeneXpert assays. Discordant test specimens were arbitrated by performing an alternative PCR assay and Sanger sequencing.

**Results:**

The TaqPath assay had a 96.7 and 99.6% positive and negative agreement respectively when compared to the Xpert Xpress test. However, after test arbitration, the three discordant specimens were arbitrated in favor of the TaqPath assay producing a positive and negative percent agreement of 100% for the TaqPath Combo Kit while the Xpress SARS-CoV-2 assay had a positive and negative percent agreement of 98.3 and 99.2% respectively.

**Conclusions:**

The TaqPath Combo Kit is a high complexity assay that compares favorably with the Xpert Xpress test and can be reliably used for the detection of SARS-CoV-2 in nasopharyngeal specimens.

## Introduction

In December 2019, an outbreak of respiratory illness caused by a previously unknown coronavirus was report in Wuhan, China [1]. The virus was subsequently identified as a novel betacoronavirus suggesting a direct transfer of the disease to humans following exposure from bats [2]. By the end of January 2020, the World Health Organization (WHO) declared the coronavirus outbreak an international public health emergency and in February the WHO named the disease as Corona Virus Disease-2019 or COVID-19 [3]. Subsequently, in early March 2020, the International Committee on Taxonomy of Viruses officially named the virus, previously called the 2019-novel Coronavirus (2019-nCoV), as SARS-CoV-2 because of the virus’ ability to cause a Severe Acute Respiratory Syndrome (SARS) in some patients [4]. The disease quickly spread globally whereby WHO officially declared COVID-19 as a pandemic on March 11, 2020 [5]. As of October 2020, over 43 million cases of COVID-19 have been documented worldwide affecting 218 countries and territories. In the United States, almost 9 million cases have been reported resulting in over 230,000 deaths [6].

On February 4, 2020, the Department of Health and Human Services (HHS) determined that COVID-19 represented a public health emergency that had a significant potential to affect national security or the health and security of United States citizens. Based on that determination, HHS subsequently declared that circumstances exist justifying the Emergency Use Authorization (EUA) of in vitro-tests for the detection and/or diagnosis of COVID-19 (https://www.fad.gov.medical-devices/emergency-use-authorizations-medical-devices/coronavirus-disease-2019-covid-19-emergency-use-authorizations-medicaldevices).

Unlike the normal FDA submission process which requires extensive experimental testing and a lengthy FDA review of the data, EUA submissions typically require a minimum of testing to validate the assay’s performance followed by a short period of time for FDA review before receiving EUA clearance [7, 8]. Given that all of these tests may not be comparable in their ability to detect SARS-CoV-2 in clinical specimens, the purpose of this study was to compare the performance of two EUA-cleared PCR assays, Cepheid’s Gene Xpert® Xpress SARS-CoV-2 test and Thermo Fisher’s TaqPath™ COVID-19 Combo Kit, to detect SARS-CoV-2 in 300 randomly selected nasopharyngeal specimens. Discordant test specimens were arbitrated using an alternative molecular method as well as Sanger sequencing.

## Materials & methods

A total of 300 randomly selected nasopharyngeal specimens were evaluated in this study that were collected from patients suspected of having COVID-19 infection between May and June of 2020. Specimens were transported to the laboratory in Universal Transport Medium (UTM) and tested using the Xpert Xpress SARS-CoV-2 assay within 2 h of collection. Of the 300 specimens, 60 were positive and 240 were negative for SARS-CoV-2 by the Xpert Xpress assay. The specimens were frozen at − 70 °C immediately after testing. Specimen were then transported on dry ice to the Masonic Medical Research Institute located in Utica, NY where they were batch tested using the TaqPath COVID-19 Combo Kit. All specimens underwent only one freeze thaw cycle prior to TaqPath testing.

### Ethical approval and consent to participate

The study was conducted using residual, de-identified specimens and no clinical or demographic information was collected. The study was conducted in accordance with the FDA guidance on *Informed Consent for* In Vitro *Diagnostic Device Studies Using Leftover Human Specimens that are Not Individually Identifiable (*April 2006). This guidance advises that informed consent is not required for this study design. The protocol was reviewed and approved by the Advarra Institutional Review Board (Columbia, MD) and determined that informed consent was not required for this study (CR0025653).

### Xpress SARS-CoV-2 assay

The Xpert Xpress SARS-CoV-2 assay is an automated in vitro diagnostic test for the qualitative detection of SARS-CoV-2 RNA using reverse transcription-PCR (RT-PCR). The Xpert Xpress SARS-CoV-2 assay is performed within a self-contained cartridge that performs extraction, amplification, and detection of amplicons if the target gene(s) are present. The cartridge also contains a Sample Process Control (SPC) and a Probe Check Control (PCC). The SPC controls for the adequate processing of the specimen and monitors for the presence of potential inhibitor(s) to the RT-PCR reaction. The PCC verifies reagent rehydration and monitors other functional activities within the cartridge.

The Xpress SARS-CoV-2 assay was performed according to the manufacturer’s instructions [9]. Basically, the specimen in UTM was mixed by inversion 5 times, a 300 μL volume was transferred to the test cartridge, and the cartridge was loaded into the Gene Xpert instrument. The assay targets the N2 and E gene sequences and, according to the manufacturer, has a LoD of 250 copies/mL. The assay has a crossing threshold (Ct) cut-off value of ≥45 cycles for negative specimens and is completed within 50 min.

### TaqPath combo kit assay

The TaqPath Combo Kit is a high complexity assay that requires a separate, stand-alone nucleic acid extraction step. The assay is performed using a 96 well microtiter tray that allows for the testing of 94 specimens as well as a positive and negative control per run. Gene amplification and amplicon detection can be performed by using any one of a number of instrument platforms, such as Applied Biosystems 7500 Fast Dx, as was used in this study. The assay targets 3 gene sequences, (N2, ORF1ab, and S genes) and is completed within 3 h.

The assay was performed according to the manufacturer’s instructions [10] by first extracting a 400 μL aliquot of specimen in UTM using the MagMAX™ Viral/Pathogen Nucleic Acid isolation kit on the KingFisher Flex Purification system (Thermo Fisher Scientific, Waltham, MA). Prior to RNA extraction, 10 μL of Proteinase K was added to each well in the KingFisher™ Deepwell 96 Plate. In addition, 10 μL of the MS2 Phage Control was added to all specimens and the Negative Control that served as an internal process control. The nucleic acid was eluted into 50 μL of Elution Solution.

For each specimen, Master Mix was prepared containing TaqPath 1-Step Multiplex Master Mix (No ROX™), COVID-19 Real Time PCR Assay Multiplex, and Nuclease-free water. 20 μL of Master Mix was dispensed into wells in a 96 well plate followed by the addition of 5 μL of eluted specimen to the appropriate well. Each run also included a SARS-CoV-2 Positive Control and a Negative Control.

Amplification was performed on the Applied Biosystems® 7500 Fast Dx Real-Time PCR Instrument (ThermoFisher Scientific, Waltham, MA). Testing was performed in batches of 94 specimens plus one negative and positive control. The results were interpreted using the Applied Biosystems™ COVID-19 Interpretive Software version 1.3. According to the manufacturer’s instructions, a specimen was considered SARS-CoV-2 positive when 2 or more SARS-CoV-2 gene targets were called positive with cycle threshold values of ≤37. The time to complete the assay for 94 specimens including the controls was approximately 3 h. According to the manufacturer, the assay has an LoD of 250 copies/mL.

### Discordant specimen testing

Specimens that produced discordant test results between the Xpress and TaqPath assays were arbitrated using two different methods: QIAstatDx™ Respiratory Panel (Qiagen, Germantown, MD) and Sanger sequencing. The QIAstatDx Respiratory Panel is a qualitative, multiplex assay that simultaneously screens for over 20 respiratory pathogens, including SARS-CoV-2. For SARS-CoV-2, the assay targets the ORF1b and E gene sequences. The assay which was performed according to the manufacturer’s instructions [11] is completed in approximately 1 h and has a LoD of 500 copies/mL. The Ct cutoff for a negative specimen is ≥37 cycles.

### Sanger sequencing method

Specimens that were discordant between the Xpert Xpress and TaqPath methods were also arbitrated using Sanger sequencing. Specimens were preamplified using a cocktail of N2, ORF1ab, and S primers that flanked the regions analyzed using the TaqPath COVID-19 Multiplex Diagnostic Solution. The size of the preamplification and sequencing amplicons are as follows; Orf1ab (369 bp, 200 bp); S-gene (354 bp, 188 bp); N-gene (357 bp, 243 bp***)*** Five microliters of specimen were combined with 2.5 μl 4x TaqPath™ 1-Step RT-qPCR Master Mix, 1 μl preamp primer cocktail (final concentration 100 nM each primer), and 1.5 μl water (10 μl total volume). Specimens were preamplified using the following PCR profile: 15 min 37 °C, 95 °C 2 min, then 25 cycles of 95 °C 3 s, 60 °C 45 s, a single final 5 min extension of 72 °C and 4 °C hold. Following preamplification, 4 μl of ExoSAP-It™ was added to the tube and incubated at 37 °C for15 min, then 80 °C for 7 min to remove the preamplification primers.

The preamplified specimens were sequenced using the Big Dye Direct protocol. Forward and reverse primers for the N2, ORF1ab, and S genes were designed with M13 forward and reverse sequencing tags. Sequencing amplicons were generated by combining 1 μl preamp reaction, 5 μl 2x Big Dye Direct PCR master mix, 1 μl N2, ORF1ab, or S M13-tagged amplification pair (final concentration 100 nM). Each reaction was set up in duplicate. Amplification was performed using the following protocol: 10 min 95 °C, then 40 cycles of 96 °C 3 s, 62 °C 15 s 68 °C 30 s, and 4 °C hold. Following PCR amplification, 2 μl Big Dye Direct Sequencing master mix and 1 μl of either Big Dye Direct M13 Forward or Reverse primer were added. Cycle sequencing was performed using the protocol: 15 min 37 °C, 80 °C 2 min, 95 °C 1 min, then 25 cycles of 96 °C 10 s, 50 °C 5 s, 65 °C 75 s, and 4 °C hold. Sequencing reactions were cleaned using the Big Dye Xterminator according to instructions supplied with the kit and analyzed on Applied Biosystems 3500xl instrument with 50 cm capillary and POP7 polymer.

Sequencing traces were analyzed using Applied Biosystems SeqScanner 2. QC Reports were generated for each trace. A trace passed if two of these three criteria was met: Trace score > 31, Contiguous read length (CRL) > 50, and QV20+ score > 50. Sequences were also aligned with the SARS-CoV-2 genome to verify identity. A specimen was called positive for the viral genome if at least one passable trace in each amplicon, in either direction, was present.

## Results

A total of 300 randomly selected, de-identified nasopharyngeal specimens were evaluated in this study of which 60 were positive and 240 were negative for SARS-CoV-2 as tested by the GeneXpert Xpress assay. Table 1 shows the comparative TaqPath test results for these specimens. Using the Xpress assay as the reference method, three TaqPath discordant test results were observed of which one specimen gave a false-positive and two produced false-negative results. The overall positive and negative percent agreements between the two assays were 96.7 and 99.6% respectively.

**Table 1:** Comparison of TaqPath and Xpert Xpress Test Results Before Arbitration Testing

Specimen Total	***N*** = 300	Xpress SARS-CoV-2	
		(+)	(−)
**TaqPathCOVID-19**	(+)	58	1
	(−)	2	239
**Statistic**	**Value**	**95% CI**	
PPA	96.67%	89.13 to 99.76%	
NPA	99.58%	96.83 to 99.79%	

Analyses of the Ct values of the target gene sequences for each of these three discordant specimens (specimen numbers 201, 284, and 288) are showed in Table 2. For the one false-positive specimen (number 201), all three TaqPath target gene sequences were detected whereas, for the two false-negative test specimens (numbers 284 and 288), only the N2 gene but not the E gene was detected by the Xpert Xpress assay. According to the manufacturer’s package insert, the detection of the N2 but not the E gene sequence is regarded as a positive test result (10).

**Table 2:** Comparative Results with Ct Values for TaqPath and Cepheid Assays for Discordant Specimens

Specimen Number	TaqPath Result	Xpress Result	TaqPath Ct Gene Values			Cepheid Ct Gene Values	
			N2	ORF lab	S	N2	E
201	Positive	Negative	33.0	33.1	32.8	N.D.^a^	N.D.
284	Negative	Positive	N.D.	N.D.	N.D.	41.9	N.D.
288	Negative	Positive	N.D.	N.D.	N.D.	40.1	N.D.

^a^*N.D*. Not detected

Arbitration testing for these three specimens was performed by using the QIAstatDx Respiratory Panel and Sanger sequencing. The results of these arbitration tests are shown in Table 3. Both arbitration test methods for the three blinded discordant specimens confirmed that the one false-positive and two false-negative TaqPath results as compared to the Xpress assay were, in fact, true positive and true negative test results. After arbitration testing, the PPA and PNA for the TaqPath assay was 100% while the PPA and PNA for the Xpress assay was 98.3 and 99.2% respectively.

**Table 3:** Summary of Arbitration Test Results Using the QIAstat-Dx Assay and Sanger Sequencing

Specimen Number	TaqPath Result	Xpress Result	QiaStat Dx Result	Sequencing Result
201	Positive	Negative	Positive (Ct value 36.7)	Positive
284	Negative	Positive	Negative	Negative
288	Negative	Positive	Negative	Negative

## Discussion

SARS-CoV-2 has emerged as a significant viral pathogen causing pandemic disease in at least 218 countries and territories resulting in over 1.2 million global deaths as of October 2020. With HHS declaring that circumstances exist justifying the EUA of in vitro diagnostics for the detection and/or diagnosis of COVID-19 [7], a large number of diagnostic tests have received EUA-clearance by the FDA [8, 12]. These diagnostic tests include gene amplification assays, antigen detection tests, and serologic methods to detect specific IgM and/or IgG antibodies. Gene amplification assays represent by far the most popular method and the greatest number of EUA-cleared tests for the detection of SARS-CoV-2 in clinical specimens.

EUA-cleared gene amplification assays may use different nucleic acid extraction methods or have no nucleic acid extraction at all, may use different methods of gene amplification, may target different gene regions specific to the virus, have different Ct criteria that mark the end of the assay, have differences in there LoDs, and other nuances that might be unique to a particular assay. Given these many differences and given that diagnostic companies need only submit minimal performance requirements to receive EUA clearance, it is possible, if not likely, that some of these assays may not be comparable in their ability to detect SARS-CoV-2 in clinical specimens [7].

This study was conducted to compare the ability of the TaqPath COVID-19 Combo Kit against the Xpert Xpress SARS-CoV-2 assay to detect SARS-CoV-2 in nasopharyngeal specimens. The Xpress SARS-CoV-2 assay was selected as the reference method because of Cepheid’s long-standing reputation in the manufacture of quality diagnostic molecular products and because the GeneXpert instrument platform and its technology is a recognized brand used in many laboratories throughout the world. The TaqPath assay was evaluated in this study because Thermo Fisher is a relatively new company entry into the field of molecular diagnostics for infectious diseases which prompted a need to evaluate the reliability of the TaqPath assay against the more established comparator method manufactured by Cepheid.

The results showed that the TaqPath Combo Kit performed slightly better than the Xpert Xpress assay for the 300 nasopharyngeal specimens evaluated in this study. After arbitration testing of the three discordant specimens by an alternative PCR method and Sanger sequencing, the TaqPath test had a 100% positive and negative percent agreement while the Xpert Xpress assay had a 98.3 and 99.2% positive and negative agreement respectively. The true positive specimen (#201) that the Xpert Xpress test called negative was positive for all three gene targets using the TaqPath assay. Two specimens (#284 and #288) that were true negatives after arbitration testing but called positive by the Xpert Xpress assay had a positive Ct values for only the N2 gene but the E gene. According to the Xpert Xpress package insert [9], a positive test is based upon the detection of the N2 gene with or without the detection of the E gene.

The TaqPath Combo kit is a high complexity assay that performed comparably to an established, moderately complex Xpert Xpress comparator method. The TaqPath COVID-19 Combo Kit is a reliable method for the detection of SARS-CoV-2 in nasopharyngeal specimens that offers the advantages of batch testing up to 94 specimens per run, a time-to-result of 3.0 h, and the potential for a lower cost per reportable.

## Data Availability

Data relative to this study are available by contacting the corresponding author. The Sangar sequencing data analyzed during this study are available in the BioStudies Database (https://www.ebi.ac.uk/biostudies/) accession number S-BSST645.
